# Annotations

**Published:** 1909-07-31

**Authors:** 


### Annotations.

### The Belfast Medical Congress.

It is just twenty-five years ago since the British Medical Association last paid an official visit to the city of Belfast. The President of the Association at that meeting was the late Professor Cumins. Now, a quarter of a century later, the President of the seventy-seventh annual meeting, which began on Friday of last week, and continues open until the last day of the present week, is the distinguished physician and therapeutist, Sir William Whitla, M.D., LL.D., Professor of Materia Medica and Therapeutics in Queen's College, Belfast, and Senior Physician to the Royal Victoria Hospital. The Association is happy in its choice of a President. Sir William Whitla, whose term of office begins with this great meeting of British medical men from all parts of the Empire, is scarcely less well known and respected by the profession beyond the shores of Erin than amongst his colleagues in Belfast and the medical practitioners of Ireland. The eight editions of his "Manual of Materia Medica," and perhaps more especially the "Dictionary of Treatment," which has passed through even more foreign than English editions, have already secured for their author a permanent reputation in medical literature and throughout the ranks of the profession. Sir William Whitla has held many presidencies in his time, and indeed as long ago as 1887 he was sectional president for therapeutics of the British Medical Association at its annual gathering; but the present headship of the 1909 Belfast meeting, in the great city with which he has been so closely and honourably identified for so many years, will probably seem to him, as it does to us, the highest of these distinctions with which his colleagues have honoured him. The proceedings of this year's meeting began on July 23 in the Assembly Hall at Belfast. At the general business meeting the chair was occupied by Dr. Sinclair White, of Sheffield, who succeeded the late Mr. Simeon Snell as president when death interrupted the yearly tenure of office; and at the subsequent representative meeting Dr. J. A. McDonald, of Taunton, presided. At the outset there was a large attendance from all parts, and it is estimated that at least 800 delegates have been present during this week's deliberations and social festivities. Fifteen scientific sections in all were arranged by the conveners of the 1909 meeting; and as we go to press their special papers and discussions are being continued daily in the Queen's College at 10 o'clock. The sections include Anatomy and Physiology; Dermatology and Electro-Therapeutics; Hæmatology and Vaccine Therapy; Diseases of Children; Navy, Army, and Ambulance; Hygiene and Public Health; Laryngology, Otology, and Rhinology; Medicine, Obstetrics and Gynæcology, Ophthalmology, Pathology, Pharmacology and Therapeutics, Psychological Medicine, Surgery, and Tropical Medicine. The programmes have given rise in many cases to interesting discussions, and these have gained much from the presence and active participation of men of eminence from foreign countries and various parts of the Empire. We hope to deal further and in some detail with the three leading professional addresses of the annual meeting, arranged for Wednesday, Thursday, and Friday of this week, and each entrusted to a leading authority upon medicine, surgery, and obstetrics respectively; and with the popular lecture on Friday evening, July 30, in the hands of the chairman of representative meetings, who has already conducted with skill and discretion the medico social, medico ethical, and medico political discussions to which these early meetings were devoted.

### A Hazardous Operation.

Some time ago the suggestion was put forward that in cases of very great hypertrophy of the heart from valvular disease, with that powerful heave against the præcordia which is familiar in these conditions, some benefit might be expected to accrue from the removal of portions of the ribs covering the hypertrophied ventricles. The operation, known under the name of cardiolysis, or alternatively as cardioplasty, has been especially advocated for adherent pericardium combined with valvular disease, for which it was originally devised by Brauer; and it is claimed that the relief to the heart by the greater freedom accorded to its movements is very considerable. Those who have worked at this subject in Germany seem to think that it is both easier and more advantageous to the patient not to remove the periosteum. In this country attention has been directed to the matter by Morison,* but the number of cases submitted to operation hitherto is said to be only fifteen. One of the dangers connected with this procedure which must be ever kept in mind is illustrated by the unfortunate outcome of an attempt to perform it on July 13 in one of the London hospitals. The patient was a boy of thirteen who had been under treatment for many months for cardiac valvular disease complicated by dropsy, following on an attack of scarlet fever. The operation of cardiolysis was suggested as a suitable one for his condition, and was agreed to by the boy's parents. Unfortunately the combination of disadvantages under which he laboured rendered him so susceptible to anæsthetisation that he died after the exhibition of no more than 2 or 3 drachms of A.C.E. mixture given on a single fold of lint upon a Schimmelbusch frame. At the subsequent post-mortem examination there was revealed mitral stenosis and regurgitation, tricuspid regurgitation, and aortic regurgitation; and the pathologist also found signs of the status lymphaticus. It certainly seems rather strange that a child suffering from the latter condition should survive an attack of scarlet fever and subsequent acute carditis, only to succumb under the influence of a minute dose of so much less formidable a poison. Be that as it may, it is evident that those for whom the operation of cardiolysis is advised are necessarily bad subjects for anæsthesia, and this fact must be very carefully taken into account by surgeons and physicians when asked for an opinion as to the possible advantages and possible risks of the operation.

* *Lancet*, July 7, 1908.

